# Epidemiology and clinical characteristics of severe non-polio enteroviruses infections: the pediatric experience of the National Institute for Infectious Diseases “Prof. Dr. Matei Balş”, a 5 year survey

**DOI:** 10.1186/1471-2334-13-S1-P102

**Published:** 2013-12-16

**Authors:** Monica Luminos, Gheorghiță Jugulete, Angelica Vişan, Magda Vasile, Anuța Bilaşco, Anca Drăgănescu, Sabina Șchiopu, Cornelia Dogaru, Mădălina Maria Merişescu, Cristina Negulescu, Osman Endis

**Affiliations:** 1 National Institute for Infectious Diseases “Prof. Dr. Matei Balş”, Bucharest, Romania; 2 Carol Davila University of Medicine and Pharmacy, Bucharest, Romania

## Background

Currently, an increase in the enteroviruses associated diseases is reported in many countries of the world. Understanding the trend of severe non-polio enteroviruses infections, in the pediatric population, becomes an important public health issue.

## Methods

We analyzed the clinical manifestations of patients aged 1 month to 14 years, with serology-confirmed non-polio enteroviral infections, who were hospitalized for at least 4 days in the National Institute for Infectious Diseases “Prof. Dr. Matei Balş”, between January 2009 and September 2013.

## Results

There were 63 laboratory-confirmed enteroviruses severe infections during this period. In our cohort 67% of the patients were males and the median age was 4.2±2.2 years of age. The average days from onset to deterioration were 4.3 days (range 1–10 days). Interestingly, more than half did not have oral ulcers, and/or characteristic skin rashes, thus making earlier diagnosis more difficult. The vast majority (92% of the patients) associated gastrointestinal manifestations.

A total of 23 patients were diagnosed with severe non-polio enterovirus aseptic meningitis, with the highest prevalence of the cases in the summer of 2012, when 18 children developed acute flaccid paralysis, 13 manifested non-specific febrile skin rashes and 9 presented with viral hepatitis.

The average time of hospitalization was 6.4 days (range 4-31 days) and all patients required interdisciplinary consults, 12 required kineto-therapy after discharge but neither one of our patients had a fatal outcome.

## Conclusion

Even in the absence of typical oral ulcers, skin rashes and/or gastrointestinal symptoms, clinicians should be alert about any flaccid paralysis episode and other neurological signs.

